# Event and Comment

**Published:** 1918-08

**Authors:** 


### DENTAL REGISTER

Vol. LXXII AUGUST, 1918 No. 8

### EVENT AND COMMENT

**Important Decisions** The famous suit in regard to the Taggart inlay patents has been decided by the District Court of Northern Illinois against the validity of the patent, and Dr. Taggart has carried the case to the higher court for retrial. The decision of course, is unsatisfactory to Dr. Taggart, and it has also precipitated a literary controversy between the editor of the *Dental Items of Interest* and the president of the Dentists Mutual Protective Alliance. This latter affair seems to promise not only wide difference of opinion as to the justice of the decision as well as of the ethics involved. The literary contestants can, of course, not expect to prejudice the ultimate legal decision, and neither will the ethics in the case be materially influenced by any literary discussion, however able may be the presentation of the opposing views. The tendency to introduce invective in place of fair reasoning, in the preliminary discussion will not help to clarify the subject, but may serve rather to create prejudices that will stand in the way of a final settlement of the questions involved, both judicially and professionally. It may be that it will be the wiser course for the profession to await the judicial settlement of the equities involved before precipitating another discussion which can serve only personal interests or prejudices.

In this connection it may be equally wise to await the further consideration of the Carr instrument decision by the upper courts. The Northern Illinois District Court has just declared the Carr patent on his set of pyorrhea instruments, invalid. This case has also been appealed to—

a higher court for retrial. The same ethical reasons apply in this case as in the Taggart case, and there is a considerable personal influence with the claimant in this case also. If personal or political influence are allowed to weigh either in arriving at judicial or professional decisions in these cases, the profession is liable to become embroiled to no good purpose. Carr and Taggart have legal rights in these cases, or they have none; and until the fundamental principle of possession is definitely settled it will be much better for the profession to await the legal decision, rather than to risk the creation of professional conditions which will do more harm than good to break down the harmonious activities of the profession in strictly ethical and professional matters of vital concern. We should do all we possibly can to encourage a strong professional organization for the better development of our profession technically and scientifically, and as a strong helper of our Government in its great struggle for the principles of liberty and righteousness.

**The Black Memorial** Two sculptors made models for the committee. The committee not feeling itself sufficiently versed in art to decide on the merits of the models presented, appointed a jury, consisting of Lorado Taft, sculptor; Charles Hutchinson, president of the Art Institute, and Charles Francis Brown, painter, to decide on the merits of the models. They decided upon the model offered by Mr. Hibbard, a Chicago sculptor of note. The committee and the family of Dr. Black examined the model and they are satisfied with it. The statue proper is of bronze. It is one-third more than life size and rests on a base of light-colored granite. It is a very imposing statue and Dr. Black's friends will be pleased with it. The likeness to Dr. Black is exceedingly good. The sculptor promised that the statue would be ready for dedication when the National Dental Association met in Chicago.

The committee asked the Park Commissioners to permit the placing of this statue in Lincoln Park, and they decided to permit this as, independent of the character it represents, it is worthy artistically to rest in this beautiful park. The statue was appropriately unveiled August 8th.

The amount of money raised for it is almost sufficient, although if there are friends who have not as yet contributed who wish to do so, their contributions will be appreciated, be they large or small.

**Another War Blunder** In order that a sufficient number of graduating dental students could be secured for the army needs a preliminary examination was asked for by the War Department one year ago, and such an examination was held for the students in the University of Michigan one month before the time for the regular final examinations, with the idea of recommending those students only who stood highest in their examination. This was a special examination and was set by the Michigan State Board of Examiners with the understanding that students who passed the best examinations, only were to be recommended to the War Department for commissions, and the remainder would be compelled to take the regular examination a month later. By an error on the part of the secretary of the Michigan Board of Examiners the report of this preliminary examination was sent to the Tabulating Committee of the National Board of Dental Examiners and was printed as the final official report for this college. As this report showed that nearly one-third of the Michigan students taking this examination had failed before the State Board it was a manifest injustice to the school, and a protest was filed with the National Board of Examiners which has corrected the mistake in a communication to the *National Dental Journal*, July issue. According to the reports of the Tabulating Committee the University of Michigan has had for the past six years only

3.3 per cent. of failures before all the examining boards of the country, and it would be a rank injustice to credit this school with 31.3 per cent. of failures, due to an official error.

